# The fear of being laughed at as additional diagnostic criterion in social anxiety disorder and avoidant personality disorder?

**DOI:** 10.1371/journal.pone.0188024

**Published:** 2017-11-27

**Authors:** Michael M. Havranek, Fleur Volkart, Bianca Bolliger, Sophie Roos, Maximilian Buschner, Ramin Mansour, Thomas Chmielewski, Katharina Gaudlitz, Josef Hättenschwiler, Erich Seifritz, Willibald Ruch

**Affiliations:** 1 Department of Psychiatry, Psychotherapy and Psychosomatics, Psychiatric Hospital, University of Zurich, Zurich, Switzerland; 2 Department of Psychology, University of Zurich, Zurich, Switzerland; 3 Privatklinik Hohenegg, Meilen, Switzerland; 4 Sanatorium Kilchberg, Kilchberg, Switzerland; 5 Centre for the Treatment of Anxiety and Depression Zurich ZADZ, Zurich, Zurich, Switzerland; 6 Neuroscience Center Zurich, University and ETH Zurich, Zurich, Switzerland; University of North Carolina at Chapel Hill, UNITED STATES

## Abstract

Social anxiety disorder (SAD) is the most common anxiety disorder and has considerable negative impact on social functioning, quality of life, and career progression of those affected. Gelotophobia (the fear of being laughed at) shares many similarities and has therefore been proposed as a subtype of SAD. This hypothesis has, however, never been tested in a clinical sample. Thus, the relationship between gelotophobia, SAD and avoidant personality disorder (APD) was investigated by examining a sample of 133 participants (64 psychiatric patients and 69 healthy controls matched for age and sex) using the Structured Clinical Interview for the Diagnostic and Statistical Manual of Mental Disorders (4th edition) and an established rating instrument for gelotophobia (GELOPH<15>). As expected, gelotophobia scores and the number of gelotophobic individuals were significantly higher among patients with SAD (*n* = 22) and APD (*n* = 12) compared to healthy controls and other psychiatric patients. Furthermore, gelotophobia scores were highest in patients suffering from both SAD and APD. In fact, all patients suffering from both disorders were also suffering from gelotophobia. As explained in the discussion, the observed data did not suggest that gelotophobia is a subtype of SAD. The findings rather imply that the fear of being laughed at is a symptom characteristic for both SAD and APD. Based on that, gelotophobia may prove to be a valuable additional diagnostic criterion for SAD and APD and the present results also contribute to the ongoing debate on the relationship between SAD and APD.

## Introduction

Social anxiety disorder (SAD, also known as social phobia) is the most common anxiety disorder and an important risk factor for secondary psychiatric illnesses such as depression or substance abuse [1]. According to the Diagnostic and Statistical Manual of Mental Disorders [2], *SAD* is described as a “marked and persistent fear of social or performance situations in which embarrassment may occur” (p. 456). This fear of being scrutinized by others may either be specific to a particular situation (e.g., public speaking), or it may be generalized to several or most social and performance situations. On a behavioral level, patients characteristically withdraw from feared social situations even though they may realize that their fears are excessive or ungrounded [2]. Physiologically, anxiety-related symptoms of arousal (like excessive blushing, sweating, trembling, palpitations and nausea) often accompany cognitive and behavioral aspects of the disorder [1]. Impairments caused by SAD have a considerable negative impact on social functioning, quality of life and career progression and tend to increase over a patient’s lifetime [3].

SAD has many close relationships and overlapping symptoms with other psychiatric disorders. In particular, there is an ongoing debate (see e.g., [4]) about the relationship between SAD and avoidant personality disorder (APD). More specifically, it is argued whether it is empirically justified that SAD and APD are conceptualized as independent entities. According to DSM-IV, *APD* (also known as anxious personality disorder) is a cluster C personality disorder characterized by a pervasive pattern of social inhibition, feelings of inadequacy, extreme sensitivity to negative evaluation, and avoidance of social interactions [2]. Increasing evidence suggests, however, that there is no clear diagnostic dividing line between SAD and APD and no separation of the two by treatment techniques (for a review see: [5]). Thus, many authors consider APD a severe form of generalized SAD rather than a separate diagnostic category. Another concept that shares many similarities with SAD is *gelotophobia* which has been defined as the fear of being laughed at and of appearing ridiculous to social partners [6]. Analogous to SAD, gelotophobia is characterized by a fear of negative evaluation, humiliation or embarrassment, a withdrawal from social situations and anxiety-related symptoms of physiological arousal [7]. However, in contrast to the relationship between SAD and APD, only few studies have examined the relationship between SAD and gelotophobia.

In a psychometric study in healthy participants, Carretero-Dios et al. [8] showed that gelotophobia (measured with the GELOPH<15>; [9]) correlated positively with scores in two instruments measuring core concepts of SAD; the Social Avoidance and Distress Scale and the Fear of Negative Evaluation scale [10]. They stated that the fear of being laughed at may reflect a more general tendency to fear negative evaluation. They added, however, that not everyone fearing negative evaluation must also fear laughter and they attributed this to differences in personal histories of those affected. Similarly, Edwards et al. [11] found a positive linear relationship between gelotophobia (measured with the GELOPH<15>) and scores in the Liebowitz Social Anxiety Scale [12] and the Brief Fear of Negative Evaluation Scale [13] in healthy participants. They argued that, from a clinical standpoint, gelotophobia seems most related to generalized SAD given that it involves a wide range of situations. Consequently, they proposed that gelotophobia may be best viewed as a specific subtype of SAD. In a further study on the association between gelotophobia and emotion-related deficits predisposing for aggression, Weiss et al. [14] found an association between gelotophobia and the propensity for SAD in healthy participants. Finally, Ritter et al. [15] hypothesized that laughter is an important social cue of acceptance or rejection and that it should therefore be subject to cognitive biases in socially anxious individuals. Congruent with this hypothesis, they found a negative laughter interpretation bias and an attention bias away from joyful/social inclusive laughter in a sample of healthy participants with high social anxiety scores. Interestingly, this effect of laughter perception in socially anxious participants was almost entirely mediated by the concept of gelotophobia. However, despite these consistent results in healthy participants, there has been no study investigating the relationship between SAD and gelotophobia in a clinical sample.

Considering the fact that still 30–40% of SAD patients cannot be helped with current treatments [1], we aimed to investigate a possible association between SAD and gelotophobia and to critically evaluate the claim that gelotophobia may be a subtype of SAD. In addition, entertaining the notion that APD is not a separate diagnosis but rather a severe form of generalized SAD, we hypothesized that if gelotophobia is associated with SAD, then it should also be associated with APD. Thus, we screened a sample of psychiatric patients with SAD and APD against several control groups including psychiatric patients with other disorders and healthy participants using an established instrument to assess gelotophobia (namely the aforementioned GELOPH<15>, [16]). In addition, we included patients with schizophrenia and with cluster A personality disorder (including schizoid, paranoid, and schizotypal personality disorders) in our control group of psychiatric patients because there were reports relating gelotophobia to these diagnoses (see e.g., [17]). We hypothesized 1.) that gelotophobia would be more prevalent in psychiatric patients compared to healthy controls and 2.) that it would be more prevalent in patients with SAD and APD compared to patients with other disorders.

## Materials and methods

### Participants and procedure

A total of one-hundred-thirty-three participants (64 psychiatric patients and 69 healthy participants matched for age and sex; 66 males and 67 females; aged 19–82 years, 40.86 ± 16.71 [*SD*]) took part in the study. The psychiatric patients were recruited by their physicians from various psychiatric institutions in the canton Zurich. The physicians were asked to inform the following patient groups about our study: patients with SAD, patients with APD, patients with schizophrenia, patients with a cluster A personality disorder and patients without any of these specific diagnoses but with an unspecific social anxiety component requiring treatment. The only exclusion criteria for the psychiatric patients was a co-occurrence of more than four physician-diagnosed psychiatric disorders. The participants of the healthy control group were recruited via online advertisements. These participants reported no past or present psychiatric or neurological diseases requiring treatment and no subjective cognitive impairments. Additional exclusion criteria for the healthy controls were regular illegal drug use and regular use of prescription drugs.

After introduction to the study and collection of demographic data, all participants were clinically interviewed by trained psychologists and medical students using the below described instruments to either assess a psychiatric diagnosis (in the group of patients) or to confirm absence of any diagnosis (in the control group). Two psychiatric patients had to be excluded because no definite diagnosis could be established and eight healthy participants were excluded because the clinical interview raised suspicion of a mental disorder. This led to a final sample of 62 psychiatric patients and 61 healthy controls, which were screened for gelotophobia using the subsequently presented questionnaire. On average, the patients in our sample suffered from 2.3 comorbid psychiatric disorders (*Median* = 2, *SD* = 1.4) and were hospitalized for 32.9 days (*Median* = 26.5, *SD* = 30.8) when they were interviewed. In order to make patients better comparable across different psychiatric disorders (particularly regarding the acute symptoms of schizophrenia), in-patients were not interviewed until their physicians declared them as stabilized and ready for discharge. The study has been carried out in accordance with the 1964 Declaration of Helsinki (as revised in 1989) and was approved by the Ethics Committee of the Canton Zurich. All participants gave written informed consent prior to the study (based on the judgments of their physicians, none of them required a legal guardian at the time when they were included in the study) and received a moderate financial compensation for participation.

### Measures

#### Psychiatric diagnoses

To assess the psychiatric diagnoses, patients were examined using the German version of the Structured Clinical Interview for DSM-IV Axis I Disorders (SCID-I) and the Structured Clinical Interview for DSM-IV Axis II Personality Disorders (SCID-II, [18]). The SCID is organized in diagnostic sections with branching tree logic and screening questions allowing an efficient and thorough mental health assessment [19]. A test-retest reliability study of the SCID for DSM-III-R showed an overall weighted κ of .61 for current and .68 for lifetime diagnoses and an inter-rater agreement of .78 [20]. In the present study, the entire SCID-I was administered, however, with a particular focus on SAD and schizophrenia. From SCID-II, only the modules for the APD and the cluster A personality disorders were administered. Both the SCID-I and the SCID-II are intended to be used to assess the presence or absence of psychiatric disorders in a binary manner (disorder present vs. not present) based on a number of diagnostic criteria specific for a given disorder.

To confirm the absence of psychiatric disorders in the control group, the similarly reliable but less time-consuming German version of the Mini International Neuropsychiatric Interview (M.I.N.I., [21]) including disorders from the tenth revision of the International Classification of Diseases and Related Health Problems (ICD-10, [22]) and from the DSM-IV was used. The M.I.N.I. is organized similarly to the SCID in diagnostic sections with branching tree logic and screening questions providing similar test-retest and inter-rater reliabilities [19, 23] and a very good convergent validity with the SCID [24] while significantly reducing the time requirements for completion [25].

#### Gelotophobia

As a measure of gelotophobia, participants filled in the German version of the GELOPH<15> [9], a standardized self-report measure of the fear of being laughed at including 15 items in a four-point answer format (from 1 =“strongly disagree” to 4 =“strongly agree”). A sample item is: “It takes me very long to recover from being laughed at.” All items are positively keyed and are averaged to form a gelotophobia score for each individual. Scores lower than 2.5 are considered as no/normal fear of being laughed at. Scores higher than 2.5 and higher than 3.0 indicate fear of being laughed at and pronounced fear of being laughed at, respectively. The GELOPH<15> is widely used in research (see e.g., [26]) and has been psychometrically evaluated and validated in several languages including German [27], showing a Cronbach’s α = .93 and a test-retest reliability of .86 and .80 for a three and a six month interval, respectively.

### Statistical analyses

Main group analyses comparing psychiatric patients with healthy controls to confirm successful matching of age and sex and to investigate potential differences in *education* (secondary vs. higher education) and *gelotophobia scores* (GELOPH<15>) were performed using independent t-tests as well as chi-square tests. A first multiple linear regression was calculated to examine whether age, sex, education, and *group* (patients vs. controls) predicted gelotophobia scores. A second multiple linear regression was used to investigate which psychiatric disorders in particular explained the gelotophobia scores. This analysis included all psychiatric diagnoses with *n*>/ = 9 as predictor variables: *SAD* (*n* = 22), *schizophrenia* (*n* = 18), *depression* (*n* = 18), *panic disorder* (*n* = 14), *APD* (*n* = 12), *addictive disorder* (*n* = 11), *cluster A personality disorder* (*n* = 9), and *other specific phobias* (n = 9) as binary variables (disorder present vs. absent, see Table 1 for additional information on the groups). Please note that these numbers add up to a total, which is higher than the total of psychiatric patients in our sample because of comorbidities. In addition, two measures of illness severity were included as covariates to assess a potential impact of illness severity on gelotophobia scores: the *number of comorbid psychiatric disorders* (in total per patient) and the *duration of the hospitalization/treatment* (days). Post-hoc t-tests were performed to confirm the results with correction for multiple comparisons (*p*_*cor*_) using the Bonferroni method. Furthermore, an independent t-test and a chi-square test were used to examine differences in gelotophobia scores between patients with one or both diagnoses of SAD and APD. Finally, a third multiple linear regression was performed to investigate the amount of variance in the gelotophobia scores that was explained by diagnoses of *SAD* and *APD* (this time as the only two predictors). Additionally, by calculating semi-partial correlation coefficients, the total variance in the gelotophobia scores found in this last regression was differentiated into the part of the variance explained by the variance common to SAD and APD in comparison to the parts of the variance explained by the variance specific to SAD and APD, respectively. Statistical analyses were conducted with SPSS (Version 20.0) and results were considered significant if *p* < .05.

**Table 1:** Overview of the sample of psychiatric patients.

Psychiatric diagnoses	Age, *mean* (*SD*)	Sex (male / female)	Education (sec. / high.)
SAD (*n* = 22)	37.14 (12.05)	12 / 10	16 / 5
Schizophrenia (*n* = 18)	36.06 (11.32)	13 / 5	17 / 1
Depression (*n* = 18)	47.18 (15.59)	5 / 13	11 / 5
Panic disorder (*n* = 14)	40.23 (13.93)	4 / 10	9 / 4
APD (*n* = 12)	35.83 (12.68)	7 / 5	11 / 1
Addictive disorder (*n* = 11)	37.73 (11.14)	9 / 2	8 / 3
Cluster A personality disorder (*n* = 9)	40.75 (11.34)	4 / 5	6 / 2
Other specific phobias (*n* = 9)	38.44 (7.97)	5 / 4	7 / 2

Social anxiety disorder is abbreviated as SAD, avoidant personality disorder is abbreviated as APD, and education is divided into secondary vs. higher education. Note that some participants did not provide any information concerning their education.

## Results

### Demography and gelotophobia comparisons of patients and controls

The group of psychiatric patients did not differ from the group of healthy participants regarding age and sex distribution. However, the number of patients with higher education was significantly lower than the number of controls possessing higher education (Table 2). Gelotophobia scores (GELOPH<15>) were significantly higher in patients compared to controls and the number of gelotophobic individuals (GELOPH<15> scores > 2.5) was significantly increased in the group of patients (Table 2). The multiple regression (*R*^*2*^ = .21, *F*(4,115) = 7.52, *p* = .000) revealed that only the variable *group* (patients vs. controls) significantly predicted gelotophobia scores (*β* = .59, *p* = .000), while age (*β* = -.00, *p* = .728), sex (*β* = .13, *p* = .261), and education (*β* = -.09, *p* = .529) did not.

**Table 2:** Demographic and gelotophobia data of healthy controls and psychiatric patients (with exception of sex, education and gelotophobia present, *means* and *SD* are shown).

Variable	Controls (*n* = 61)	Patients (*n* = 62)	*t-test/Χ*^*2*^	*df*	*p*
Age, *mean* (*SD*)	41.1 (19.3)	40.4 (13.2)	0.2	106.0	.819
Sex (male / female)	30 / 31	33 / 29	0.2	1	.720
Education (secondary / higher)	21 / 39	44 / 16	17.6	1	**.000**
GELOPH<15> score, *mean* (*SD*)	1.7 (0.5)	2.3 (0.7)	-5.3	110.7	**.000**
Gelotophobia present (yes / no)	6 / 55	27 / 35	17.8	1	**.000**

*Gelotophobia present* refers to individuals with GELOPH<15> scores higher than 2.5

### Gelotophobia comparisons of the different psychiatric disorders

Comparing the psychiatric disorders in the second multiple linear regression revealed that only *SAD* and *APD* significantly predicted gelotophobia scores, while all other disorders and the covariates *number of comorbid disorders* and *duration of hospitalization* did not (Table 3). Post-hoc t-tests confirmed that gelotophobia scores differed significantly between participants with (*M* = 2.8, *SD* = 0.7) and without (*M* = 1.8, *SD* = 0.6) SAD (*t*(121) = 7.17, *p*_*cor*_ = .000) and between participants with (*M* = 2.9, *SD* = 0.7) and without (*M* = 1.9, *SD* = 0.7) APD (*t*(121) = 5.34, *p*_*cor*_ = .000).

**Table 3:** Multiple linear regression predicting GELOPH<15> scores in patients and controls (*n* = 123).

Variable	*B*	*SE B*	*β*
SAD	0.61	0.20	.33**
Schizophrenia	-0.18	0.22	-.09
Depression	0.42	0.22	.21
Panic disorder	0.36	0.29	.16
APD	0.66	0.26	.27*
Addictive disorder	0.25	0.25	.10
Cluster A PD	0.36	0.39	.13
Other specific phobias	0.40	0.27	.14
Number of comorbid D	-0.04	0.14	-.09
Duration of hospitalization	0.00	0.02	-.01
*R*^*2*^		.45	
*F*		9.13***	

SAD = social anxiety disorder, APD = avoidant personality disorder, Cluster A PD = cluster A personality disorder, Number of comorbid D = number of comorbid psychiatric disorders

*p < .05,

**p < .01,

***p < .001

### Gelotophobia comparisons of SAD and APD

**Fig 1:**
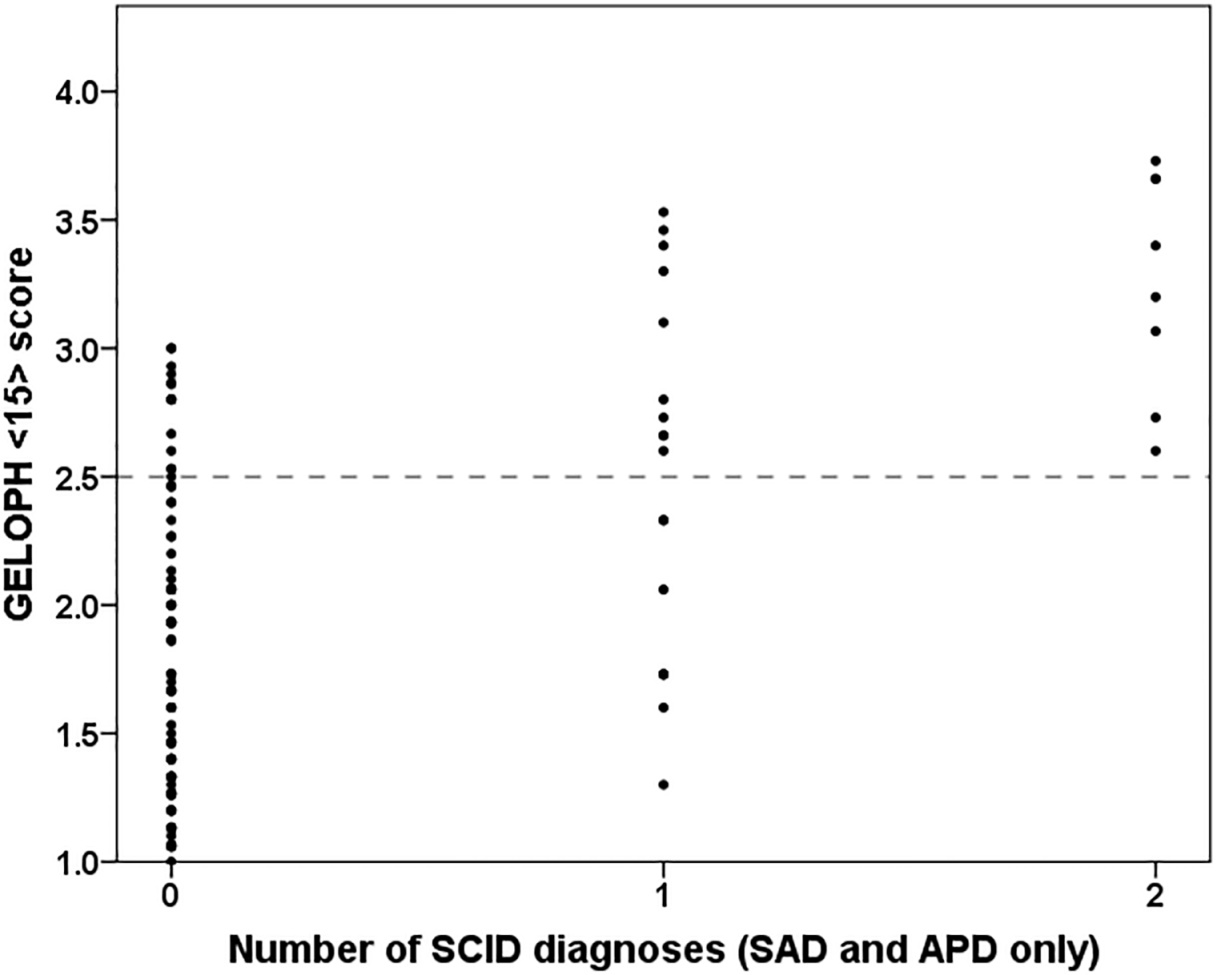
Scatter plot depicting the relationship between the number of SCID diagnoses of SAD and APD and the GELOPH<15> score. The dashed horizontal line illustrates the cut-off for gelotophobia (GELOPH<15> score > 2.5).

Eight out of twelve cases of APD co-occurred with SAD (66.67%). Because of this, gelotophobia differences between patients with only one disorder (14 patients with SAD and four patients with APD) and patients with both disorders (eight patients) were examined. In patients suffering from both disorders (*M* = 3.3, *SD* = 0.4), gelotophobia scores were significantly higher (*t*(24) = 2.80, *p* = .010) compared to patients suffering only from one disorder (*M* = 2.5, *SD* = 0.7). Similarly, in patients with both disorders, the number of gelotophobic individuals was significantly higher than in patients with only one disorder (*X*^*2*^(1,26) = 5.14, *p* = .023). In fact, all patients suffering from both disorders were classified as gelotophobic as well (see Fig 1). Finally, the third multiple linear regression predicting gelotophobia scores using *SAD* and *APD* as the only predicting diagnoses revealed that SAD (*β* = .44, *p* = .000) and APD (*β* = .25, *p* = .002) explained 35.10% of the variance in gelotophobia scores (*F*(2,122) = 32.38, *p* = .000). Calculating the semi-partial correlation coefficients showed additionally that of this total variance explained by SAD and APD, 13.87% was predicted by the variance common to SAD and APD, whereas 15.97% and 5.26% was predicted by the variance specific to SAD and APD, respectively.

## Discussion

This was the first study investigating the relationship between gelotophobia, SAD, and APD in a clinical sample of psychiatric patients. By screening psychiatric patients and healthy controls with the GELOPH<15>, we demonstrated that gelotophobia scores and the number of gelotophobic individuals were higher in patients compared to controls and higher in patients with SAD and APD compared to patients with other psychiatric disorders. In addition, gelotophobia scores were higher in patients suffering from both SAD and APD compared to patients with only one of these disorders. In fact, even though we also found gelotophobic individuals without SAD and APD, we found no patients suffering from both SAD and APD that were not gelotophobic as well.

Our results are consistent with previous studies investigating the relationship between social anxiety and gelotophobia in healthy participants. Carretero-Dios et al. [8] found that GELOPH<15> scores correlated positively with scores in the Social Avoidance and Distress Scale (*r* = .64) and in the Fear of Negative Evaluation scale (*r* = .52), while Edwards et al. [11] reported a positive correlation between GELOPH<15> scores and scores in the Liebowitz Social Anxiety Scale (*r* = .67) and in the Brief Fear of Negative Evaluation Scale (*r* = .70). Based on their results, Edwards et al. [11] proposed that gelotophobia may be a specific subtype of generalized SAD and suggested to test this hypothesis in a clinical sample. We investigated this particular notion in the present study in a sample of psychiatric patients and healthy controls. However, our results do not support their hypothesis because despite a large diagnostic overlap, we also found gelotophobic individuals (patients and healthy participants) without SAD or APD and SAD together with APD explained only 35.10% of the variance in gelotophobia scores. On the other hand, we did find no individuals without gelotophobia in patients suffering from both SAD and APD. Thus based on our results, it seems that rather than a subtype of generalized SAD, gelotophobia could be seen as a symptom of SAD and APD, which is necessary but not sufficient in patients suffering from both disorders. In other words, we propose that if a patient has both SAD and APD comorbidly, he must also be gelotophobic. However, not every gelotophobic individual must also have SAD and APD. In our opinion, these findings imply that the fear of being laughed at (as a potential symptom of SAD and APD) could be compared to other specifically feared social or performance situations (such as for example the fear of speaking in public). Not all SAD patients are afraid of speaking in public. However, as a specific SAD patient progresses in severity to a generalized SAD (or even to a generalized SAD with a comorbid APD) the number of feared social or performance situations increases as well (see [28]) and with it increases the probability of fearing any specific single social or performance situation such as speaking in public. Accordingly, the same logic may be applied to gelotophobia. The more social or performance situations an individual (e.g., an SAD patient) fears, the more likely it is that the fear of being laughed at is among them.

Our sample of psychiatric patients was characterized by a large number of comorbidities, especially concerning the disorders of interest, whereby eight out of twelve (66.67%) diagnosed APD patients also qualified for a diagnosis of SAD. These results are according to expectations, particularly regarding the ongoing debate on the relationship between SAD and APD. Several authors (see e.g., [5]) argued that APD should be seen as a severe form of generalized SAD rather than as a separate diagnostic entity because there is no clear diagnostic dividing line and no separation by treatment techniques between the two diagnoses. Because of that, we were especially interested in the triangle relationship between SAD, APD, and gelotophobia as well. On the one hand, we found that patients suffering from both SAD and APD (eight patients) showed higher gelotophobia scores than patients with only one of the two disorders (14 patients with SAD and four patients with APD). On the other hand, we observed that of the total variance in gelotophobia scores explained by SAD and APD (35.10%), a large part was admittedly explained by the variance common to both SAD and APD (13.87%). However, still more than half of the total variance in gelotophobia scores was explained by the variance specific to SAD and the variance specific to APD (15.97% and 5.26%, respectively). The former finding of increasing gelotophobia symptom severity in patients with both SAD and APD compared to patients with only SAD (see also Fig 1) seems to support the view that SAD and APD are part of the same continuum rather than two separate diagnostic entities. However, the latter finding seems to suggest that at least regarding gelotophobia, there is nevertheless some specific and relevant variance to both diagnostic categories.

If we assume that gelotophobia is a symptom of SAD and APD and not a subtype, it would be possible that this symptom occurs in other psychiatric disorders as well. In fact, there is evidence from previous studies pointing to a connection between gelotophobia and schizophrenia as well as cluster A personality disorders. In the only other clinical study on gelotophobia presently available, Forabosco et al. [17] also found increased GELOPH<15> scores in psychiatric patients compared to healthy controls. However, in their study, this effect was attributable to patients with personality disorders and patients with schizophrenia. Unfortunately, they did not provide information on the types of personality disorder that were included in their sample. Because of this, we do not know how many APD were part of their group of personality disorder. However, concerning patients with schizophrenia, we were not able to replicate their findings. On the contrary, our schizophrenic patients had the lowest gelotophobia scores among all our psychiatric patients. We can only speculate on the reasons for this contrasting result. One explanation might be that Forabosco et al. [17] tested their schizophrenic patients during an acute psychotic episode, while we deliberately tested our patients after the acute symptoms have been alleviated and the patients have been stabilized. We chose this procedure because our pilot study revealed that acute psychotic patients did not sufficiently understand the questionnaire items.

In addition, three studies in healthy participants by Weiss et al. [14] and Papousek et al. [29, 30] not only found an association between gelotophobia and a propensity for SAD but also between gelotophobia and a propensity for cluster A personality disorders. Again, we can only speculate on why we did not find an association between gelotophobia and cluster A personality disorders in our sample. The most likely reason for this discrepancy probably is our small sample size of patients with cluster A personality disorders in combination with the large number of comorbidities present. On the other hand, it must be pointed out that Weiss et al. [14] as well as Papousek et al. [29, 30] used the reverse approach for participant selection compared to us. They prescreened healthy participants for gelotophobia and then characterized them for clinical propensities, while we preselected actual clinical patients and then assessed their gelotophobia scores. These different approaches may impede the comparability of our results.

Our study has two important limitations worth mentioning. The first and obvious one is the small sample size of our group of psychiatric patients overall and of specific subgroups of patients (such as the cluster A personality disorders) in particular. This fact additionally increases the problem of large numbers of comorbidities which are characteristic for psychiatric samples and have been found in our particular sample as well. Because of this combination of a small sample size combined with several comorbidities, the relevance of some subgroups of patients may have been underestimated in our analyses. A second limitation is the fact that we did simply define our psychiatric disorders as binary variables (disorder present vs. absent) without additionally assessing the symptom severity of the psychiatric patients in our sample. Particularly concerning our contrasting results compared to some previous studies a further characterization of symptom severity would have been interesting. Future studies should therefore use larger clinical samples as well as additional measurement instruments to confirm or challenge our results.

In conclusion, we presented evidence for a relationship between gelotophobia, SAD, and APD in a clinical sample of psychiatric patients. We showed that gelotophobia scores and the number of gelotophobic individuals were highest among patients with SAD and APD and that all patients suffering from both SAD and APD were also suffering from gelotophobia. If these findings can be replicated in other clinical samples, gelotophobia might prove to be an overlooked symptom of SAD and APD and could be used as an additional diagnostic criterion for the two disorders.
